# Crystal structure of glutamyl-tRNA synthetase from *Helicobacter pylori*

**DOI:** 10.1107/S2053230X24011099

**Published:** 2024-11-27

**Authors:** Dylan E. Davis, Jesuferanmi P. Ayanlade, David T. Laseinde, Sandhya Subramanian, Hannah Udell, Donald J. Lorimer, David M. Dranow, Thomas E. Edwards, Peter J. Myler, Oluwatoyin A. Asojo

**Affiliations:** a Dartmouth Cancer Center, One Medical Center Drive, Lebanon, NH03756, USA; b College of Arts and Science Dartmouth College Hanover NH03755 USA; c College of Arts and Sciences University of Southern Mississippi Hattiesburg MS39406 USA; d Center for Global Infectious Disease Research Seattle Children’s Research Institute 307 Westlake Avenue, North Suite 500 Seattle WA98109 USA; e Seattle Structural Genomics Center for Infectious Diseases, Seattle, Washington, USA; f UCB BioSciences, Bainbridge Island, WA98110, USA; University of York, United Kingdom

**Keywords:** undergraduate education and training, glutamyl-tRNA synthetase, cancer, infectious diseases, gastric ulcers, Seattle Structural Genomics Center for Infectious Disease

## Abstract

*Helicobacter pylori* is one of the most common bacterial infections; over two-thirds of the world’s population is infected by early childhood. Persistent *H. pylori* infection results in gastric ulcers and cancers. Due to drug resistance, there is a need to develop alternative treatments to clear *H. pylori*. The Seattle Structural Genomics Center for Infectious Disease (SSGCID) conducts structure–function analysis of potential therapeutic targets from *H. pylori*. Glutamyl-tRNA synthetase (GluRS) is essential for tRNA aminoacylation and is under investigation as a bacterial drug target. The SSGCID produced, crystallized and determined the apo structure of *H. pylori* GluRS (*Hp*GluRS). *Hp*GluRS has the prototypical bacterial GluRS topology and has similar binding sites and tertiary structures to other bacterial GluRS that are promising drug targets. Residues involved in glutamate binding are well conserved in comparison with *Pseudomonas aeruginosa* GluRS (*Pa*GluRS), which has been studied to develop promising new inhibitors for *P. aeruginosa*. These structural similarities can be exploited for drug discovery and repurposing to generate new antibacterials to clear persistent *H. pylori* infection and reduce gastric ulcers and cancer.

## Abstract 2

Persistent *H. pylori* infection causes gastric ulcers and cancer, and multidrug resistance is increasing globally. The 2.5 Å resolution structure of *H. pylori* glutamyl-tRNA synthetase was determined as a first step towards rational drug discovery.

## 1. Introduction

*Helicobacter pylori* is a Gram-negative, flagellated and helical bacterium that was first discovered in 1982 in the guts of patients with gastric ulcers and gastritis, establishing the causative role of *H. pylori* in the development of stomach ulcers (Warren & Marshall, 1983 ▸). According to the Centers for Disease Control, about two-thirds of the world’s population is infected by *H. pylori* by early childhood, and transmission occurs through the fecal–oral, oral–oral or gastric–oral routes. The spiral-shaped *H. pylori* colonizes the gastric epithelium, and persistent *H. pylori* infection can lead to peptic ulcers, gastritis and gastric cancer (Malfertheiner *et al.*, 2022 ▸). *H. pylori* causes peptic ulcers that involve the formation of sores in the stomach lining or part of the upper small intestine, while significantly increasing the risk of developing gastric cancer and gastric mucosa-associated lymphoid tissue (MALT) lymphoma (Crowe, 2019 ▸). The pathophysiology of *H. pylori* depends on environmental factors and the host immune system. Increasing antibiotic resistance of *H. pylori* is a global cause for concern, and *H. pylori* is currently treated with bismuth or non-bismuth quadruple therapy for 14 days as a first-line treatment in regions with high clarithromycin or metronidazole resistance (Boyanova *et al.*, 2023 ▸; Suzuki *et al.*, 2019 ▸). *H. pylori* is one of the major priorities for structure-based drug discovery by the Seattle Structural Genomics Center for Infectious Disease (SSGCID). Towards these ends, the SSGCID selected glutamyl-tRNA synthetase (GluRS) from *H. pylori* (*Hp*GluRS) as a drug-repurposing and drug-identification target. GluRS catalyzes tRNA aminoacylation: the covalent linkage of glutamate to tRNA. Aminoacyl-tRNA synthases (aaRSs) are enzymes that catalyze the attachment of amino acids to tRNA molecules during transcription (aminoacylation), which is a crucial step in protein synthesis. GluRS and other aminoacyl-tRNA synthetases are crucial for bacterial survival and are promising targets for drug discovery for infectious diseases (Kwon *et al.*, 2019 ▸; Lee *et al.*, 2018 ▸; Moen *et al.*, 2017 ▸; Narsimulu *et al.*, 2024 ▸; Hu *et al.*, 2018 ▸; Brooks *et al.*, 2022 ▸). Here, we report the production, crystallization and 2.5 Å resolution structure of *Hp*GluRS.

## 2. Materials and methods

### 2.1. Macromolecule production

*Hp*GluRS was cloned, expressed and purified using established protocols (Stacy *et al.*, 2011 ▸; Serbzhinskiy *et al.*, 2015 ▸; Rodríguez-Hernández *et al.*, 2023 ▸). The gene for *Hp*GluRS (UniProt B5Z6J9) encoding amino acids 1–463 was PCR-amplified from genomic DNA using the primers shown in Table 1 ▸. The gene was ligated into the expression vector BG1861 to generate plasmid DNA. Chemically competent *Escherichia coli* BL21(DE3)R3 Rosetta cells were transformed with the plasmid DNA. The plasmid-containing His-*Hp*GluRS cells were tested for expression, and 2 l of culture was grown using auto-induction medium (Studier, 2005 ▸) in a LEX Bioreactor (Epiphyte Three) as described previously (Serbzhinskiy *et al.*, 2015 ▸). The expression clone can be requested online at https://www.ssgcid.org/available-materials/expression-clones/.

*Hp*GluRS was purified in two steps: an immobilized metal (Ni^2+^) affinity chromatography (IMAC) step and size-exclusion chromatography (SEC) on an ÄKTApurifier 10 (GE Healthcare) using automated IMAC and SEC programs (Serb­zhinskiy *et al.*, 2015 ▸). Briefly, thawed bacterial pellets (25 g) were lysed by sonication in 200 ml lysis buffer [25 m*M* HEPES pH 7.0, 500 m*M* NaCl, 5%(*v*/*v*) glycerol, 0.5%(*w*/*v*) CHAPS, 30 m*M* imidazole, 10 m*M* MgCl_2_, 1 m*M* TCEP, 250 mg ml^−1^ AEBSF, 0.025%(*w*/*v*) sodium azide]. After sonication, the crude lysate was treated with 20 µl (25 units ml^−1^) of Benzonase and incubated while mixing at room temperature for 45 min. The lysate was clarified by centrifugation at 10 000*g* for 1 h using a Sorvall centrifuge (Thermo Scientific). The treated supernatant was then passed over an Ni–NTA HisTrap FF 5 ml column (GE Healthcare) which had been pre-equilibrated with loading buffer [25 m*M* HEPES pH 7.0, 500 m*M* NaCl, 5%(*v*/*v*) glycerol, 30 m*M* imidazole, 1 m*M* TCEP, 0.025%(*w*/*v*) sodium azide]. The column was washed with 20 column volumes (CV) of loading buffer and was eluted with elution buffer [25 m*M* HEPES pH 7.0, 500 m*M* NaCl, 5%(*v*/*v*) glycerol, 30 m*M* imidazole, 1 m*M* TCEP, 0.025%(*w*/*v*) sodium azide, 250 m*M* imidazole] over a 7 CV linear gradient. Peak fractions were pooled, concentrated to 5 ml and loaded onto a Superdex 75 column (GE Healthcare) equilibrated with running buffer [20 m*M* HEPES pH 7.0, 300 m*M* NaCl, 5%(*v*/*v*) glycerol, 1 m*M* TCEP]. The peak fractions were collected and analyzed using SDS–PAGE. *Hp*GluRS eluted as a symmetrical monodisperse peak accounting for >90% of the protein product at a molecular mass of ∼50 kDa, suggesting purification as a monomer (the expected monomer molecular weight was 54 kDa). The peak fractions were pooled and concentrated to 62.8 mg ml^−1^ using an Amicon filtration system (Millipore). Aliquots of 110 µl were flash-frozen in liquid nitrogen and stored at −80°C until use. Purified *Hp*GluRS can be requested online at https://www.ssgcid.org/available-materials/ssgcid-proteins/.

### 2.2. Crystallization

*Hp*GluRS was crystallized at 290 K using sitting-drop vapor diffusion. Briefly, 20.6 mg ml^−1^ protein was mixed in a 1:1 ratio with the precipitant solution as described in Table 2 ▸. Before data collection, the crystals were harvested and cryoprotected with 15%(*v*/*v*) ethylene glycol (Table 2 ▸).

### 2.3. Data collection and processing

Data were collected at 100 K on beamline 21-ID-G at the Advanced Photon Source (APS), Argonne National Laboratory (Table 3 ▸). Data were integrated with *XDS* and reduced with *XSCALE* (Kabsch, 2010 ▸). Raw X-ray diffraction images have been stored at the Integrated Resource for Reproducibility in Macromolecular Crystallography at https://www.proteindiffraction.org.

### 2.4. Structure solution and refinement

The structure of *Hp*GluRS was determined by molecular replacement with *Phaser* (McCoy *et al.*, 2007 ▸) from the *CCP*4 suite of programs (Collaborative Computational Project, Number 4, 1994 ▸; Krissinel *et al.*, 2004 ▸; Winn *et al.*, 2011 ▸; Agirre *et al.*, 2023 ▸) using PDB entry 2ja2 (G. P. Bourenkov, N. Strizhov, L. A. Shkolnaya, M. Bruning & H. D. Bartunik, unpublished work) as the search model. The structure was refined using *Phenix* (Liebschner *et al.*, 2019 ▸). The structure quality was checked using *MolProbity* (Williams *et al.*, 2018 ▸). Data-reduction and refinement statistics are shown in Table 4 ▸. Coordinate and structure factors have been deposited with the Worldwide PDB (wwPDB) as entry 6b1p. The accuracy of the ligands and waters was also checked with the *CheckMyBlob* server (Kowiel *et al.*, 2019 ▸; https://checkmyblob.bioreproducibility.org/server/).

## 3. Results and discussion

**Figure 1:**
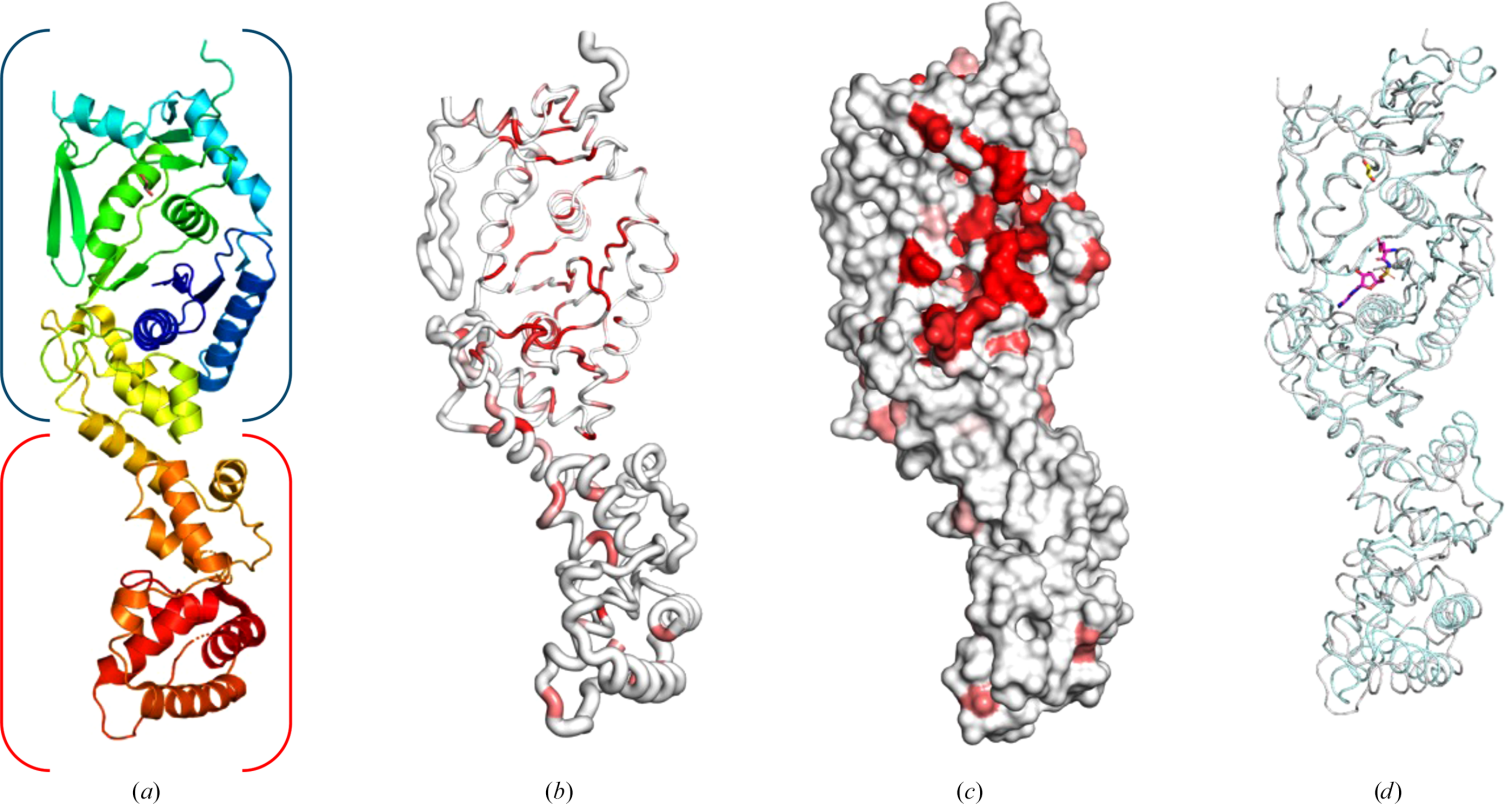
Overall structure of *Hp*GluRS. (*a*) The *Hp*GluRS monomer in rainbow colors from blue at the N-terminus to red at the C-terminus. The glutamate-binding site is indicated in blue parentheses, while the tRNA-binding site is indicated in red parentheses. (*b*) Ribbon diagram calculated by *ENDScript*. The circumference of the ribbon (sausage) represents relative structural conservation compared with other GluRS structures (these structures are indicated in Supplementary Fig. S1). Thinner ribbons represent more highly conserved regions. In comparison, thicker ribbons represent less conserved regions, and the ribbon is colored by sequence conservation, with red indicating identical residues. (*c*) The solvent-accessible surface area of *Hp*GluRS is colored by sequence conservation, with red indicating identical residues. (*d*) Superposed *Hp*GluRS (gray) with *Thermotoga maritima* GluRS (*Tm*GluRS; PDB entry 3afh, cyan; Ito *et al.*, 2010 ▸) reveals a conserved prototypical GluRS topology; a glutamyl-AMP analog (magenta sticks) is sitting in the glutamate-binding site. An ethylene glycol molecule from the cryoprotectant is shown as yellow sticks. (*a*)–(*d*) are shown in the same orientation.

Size-exclusion chromatography data suggest that *Hp*GluRS assembles as a monodisperse monomer in solution with a calculated molecular weight of ∼50 kDa, close to the theoretical mass of 54.3 kDa. One *Hp*GluRS monomer is present in the asymmetric unit, which also appears to correspond to the biological unit (Fig. 1 ▸*a*). *Hp*GluRS has the prototypical bacterial GluRS topology containing N-terminal tRNA synthetase class I (E and Q) catalytic and C-terminal anticodon-binding domains. The only ligand in this structure is an ethylene glycol from the crystallization solution (Fig. 1 ▸*a*).

**Figure 2:**
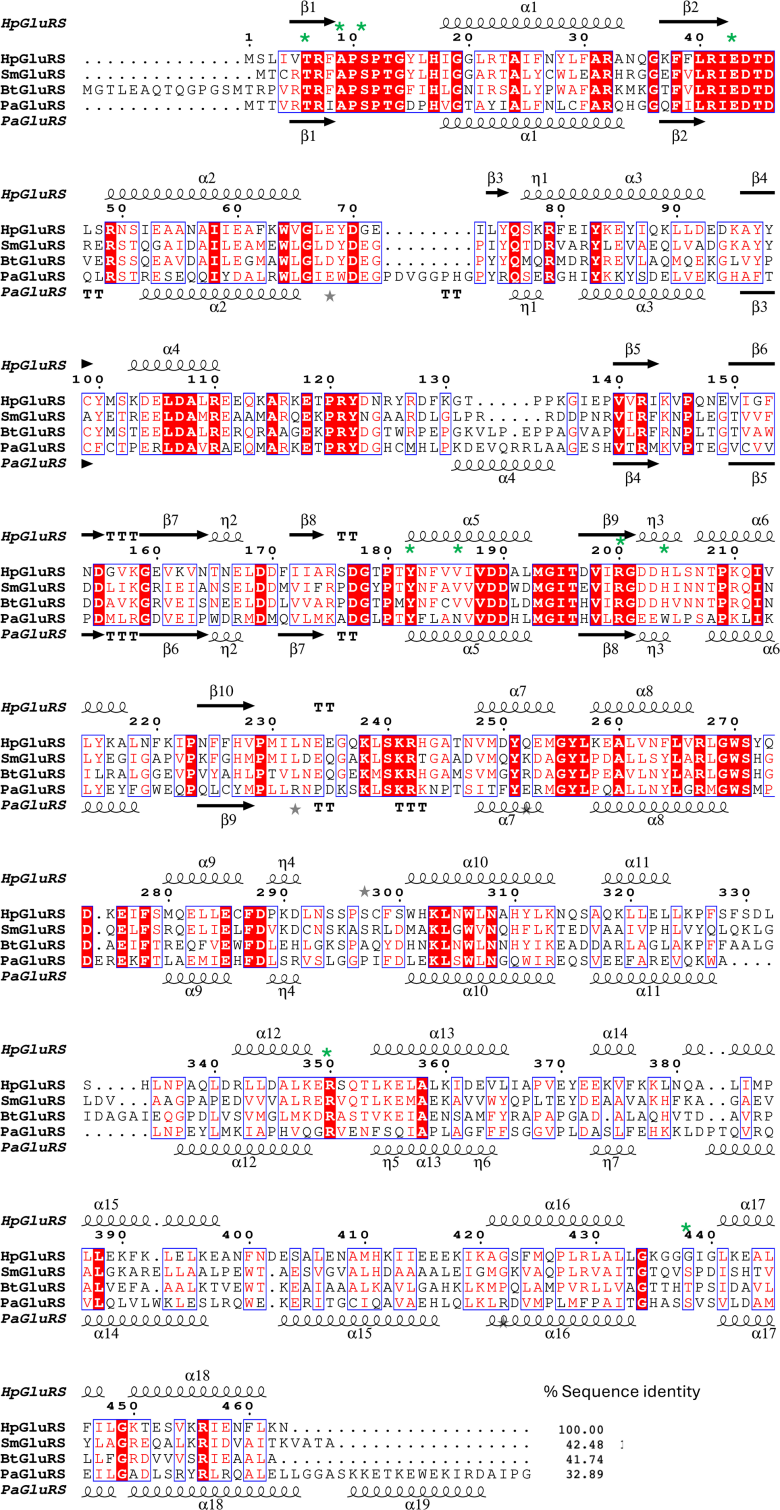
Primary-sequence alignment of *Hp*GluRS (PDB entry 6b1p), *Sm*GluRS, *Bt*GluRS and *Pa*GluRS (PDB entry 5tgt). Residues involved in glutamate binding are indicated by green asterisks. The secondary-structure elements are as follows: α-helices are shown as large coils, 3_10_-helices are shown as small coils labeled η, β-strands are shown as arrows labeled β and β-turns are labeled TT. Identical residues are shown on a red background, with conserved residues in red and conserved regions in blue boxes. This figure was generated using *ESPript* 3.0 (Gouet *et al.*, 1999 ▸, 2003 ▸). Additional structural details and alignments are shown in Supplementary Fig. S1.

*ENDScript* (Gouet *et al.*, 2003 ▸; Robert & Gouet, 2014 ▸) analysis (Figs. 1 ▸*b* and 1 ▸*c*) reveals that *Hp*GluRS is a prototypical bacterial GluRS. *PDBeFold* (https://www.ebi.ac.uk/msd-srv/ssm/) analysis (Krissinel & Henrick, 2004 ▸) using a default threshold of 70% validated the *ENDScript* analysis (Supplementary Fig. S2). *ENDScript* analysis confirms that *Hp*GluRS shares significant secondary-structural similarity with other bacterial GluRS and other aminoacyl-tRNA synthetases, including some that have shown promise as drug targets (Supplementary Fig. S1). *ENDScript* analysis reveals the closest structural neighbors of *Hp*GluRS to include *Burkholderia thailandensis* GluRS (*Bt*GluRS; PDB entry 4g6z; Moen *et al.*, 2017 ▸), which shares 42.5% sequence identity with *Hp*GluRS, and *Stenotrophomonas maltophilia* GluRS (*Sm*GluRS; PDB entry 7k86; Seattle Structural Genomics Center for Infectious Disease, unpublished work), with 41.7% sequence identity to *Hp*GluRS (Supplementary Fig. S1). These are also revealed to be close structural neighbors by *PDBeFold* (Supplementary Table S1). The regions of most significant structural similarity are in the N-terminal domain (Fig. 2 ▸ and Supplementary Fig. S1), which is considerably thinner in the *ENDScript* sausage plot (Fig. 1 ▸*b*). Additional structural comparisons and phylogenetic analysis are detailed in Supplementary Figs. S1–S4.

The sizeable accessible glutamate-binding site in the N-terminal tRNA synthetase binding domain of *Hp*GluRS is evident in the surface plot (Fig. 1 ▸*c*). The glutamate-binding region is highly conserved, as indicated by a red color in the ribbon and surface plots (Figs. 1 ▸*c* and 1 ▸*d*). Bacterial GluRSs, like other aminoacyl-tRNA synthetases, are promising antimicrobial targets (Kwon *et al.*, 2019 ▸; Lee *et al.*, 2018 ▸; Moen *et al.*, 2017 ▸; Pang *et al.*, 2021 ▸). *Pseudomonas aeruginosa* GluRS (*Pa*GluRS; PDB entry 5tgt; Seattle Structural Genomics Center for Infectious Disease, unpublished work), the glutamate-binding cavity of which has been probed to develop promising inhibitors for *P. aeruginosa* (Escamilla *et al.*, 2020 ▸; Hu *et al.*, 2015 ▸, 2018 ▸), shares considerable structural similarity with *Hp*GluRS despite having less than 33% sequence identity (Fig. 2 ▸). More importantly, the amino acids involved in glutamate binding, indicated by green asterisks, are well conserved (Fig. 2 ▸). These glutamate-binding pockets are also conserved in other bacterial GluRSs (Supplementary Fig. S1), suggesting that structure-based and rational inhibitor design for *Pa*GluRS and other bacterial GluRSs may be a starting point for *Hp*GluRS.

## 4. Conclusion

The production, crystallization and 2.5 Å resolution structure of *H. pylori* glutamyl-tRNA synthetase (*Hp*GluRS) reveals a prototypical bacterial GluRS with well conserved glutamate-binding cavities. The structural similarity to the well studied *P. aeruginosa* GluRS and lessons learned from other bacterial GluRSs may accelerate the development of new inhibitors for *H. pylori*, a globally important bacterium that causes gastric ulcers and cancer.

## Supplementary Material

PDB reference: glutamyl-tRNA synthetase, 6b1p

Supplementary figures. DOI: 10.1107/S2053230X24011099/ir5036sup1.pdf

## Figures and Tables

**Table 1:** Macromolecule-production information

Source organism	*Helicobacter pylori* (strain G27)
DNA source	Nina Salama, FHCRC
Forward primer	5′-CTCACCACCACCACCACCATATGAGTTTGATCGTTACGCGCTTC-3′
Reverse primer	5′-ATCCTATCTTACTCACTTAGTTTTTCAAAAAATTTTCTATTCTTTTGA-3′
Expression vector	BG1861
Expression host	*Escherichia coli* BL21(DE3)R3 Rosetta
Complete amino-acid sequence of the construct produced	MAHHHHHHMSLIVTRFAPSPTGYLHIGGLRTAIFNYLFARANQGKFFLRIEDTDLSRNSIEAANAIIEAFKWVGLEYDGEILYQSKRFEIYKEYIQKLLDEDKAYYCYMSKDELDALREEQKARKETPRYDNRYRDFKGTPPKGIEPVVRIKVPQNEVIGFNDGVKGEVKVNTNELDDFIIARSDGTPTYNFVVIVDDALMGITDVIRGDDHLSNTPKQIVLYKALNFKIPNFFHVPMILNEEGQKLSKRHGATNVMDYQEMGYLKEALVNFLVRLGWSYQDKEIFMQELLECFDPKDLNSSPSCFSWHKLNWLNAHYLKNQSAQKLLELLKPFSFSDLSHLNPAQLDRLLDALKERSQTLKELALKIDEVLIAPVEYEEKVFKKLNQALIMPLLEKFKLELKEANFNDESALENAMHKIIEEEKIKAGSFMQPLRLALLGKGGGIGLKEALFILGKTESVKRIENFLKN

**Table 2:** Crystallization

Method	Vapor diffusion, sitting drop
Plate type	Tray 101-d6, 96-well plates
Temperature (K)	290
Protein concentration (mg ml^−1^)	20.6
Buffer composition of protein solution	20 m*M* HEPES pH 7.0, 300 m*M* NaCl, 5% glycerol, 1 m*M* TCEP
Composition of reservoir solution	0.2 *M* ammonium citrate dibasic, 20%(*w*/*v*) PEG 3350
Volume and ratio of drop	0.4 µl, 1:1
Volume of reservoir (µl)	80
Composition of cryoprotectant solution	0.17 *M* ammonium citrate dibasic, 17%(*w*/*v*) PEG 3350, 15% ethylene glycol

**Table 3:** Data collection and processing Values in parentheses are for the outer shell.

Diffraction source	Beamline 21-ID-G, APS
Temperature (K)	100
Detector	MAR Mosaic 300 mm CCD
Space group	*P*2_1_2_1_2_1_
*a*, *b*, *c* (Å)	203.05, 44.60, 54.12
α, β, γ (°)	90, 90, 90
Resolution range (Å)	43.56–2.50 (2.56–2.50)
Total No. of reflections	103691
Completeness (%)	99.80 (100)
Multiplicity	5.84 (6.15)
〈*I*/σ(*I*)〉	15.19 (3.18)
*R*_r.i.m._	0.076 (0.58)
Overall *B* factor from Wilson plot (Å^2^)	50.48

**Table 4:** Structure solution and refinement Values in parentheses are for the outer shell.

Resolution range (Å)	43.56–2.50 (2.57–2.50)
Completeness (%)	97.0
No. of reflections, working set	17249 (1172)
No. of reflections, test set	1740 (135)
Final *R*_cryst_	0.234 (0.313)
Final *R*_free_	0.281 (0.381)
No. of non-H atoms	
Protein	3215
Ligand	4
Solvent	27
Total	3246
R.m.s. deviations	
Bond lengths (Å)	0.003
Angles (°)	0.502
Average *B* factors (Å^2^)	
Protein	63.4
Ligand	66.3
Water	50.7
Ramachandran plot	
Most favored (%)	96.7
Allowed (%)	3.3
