# Supplementary material for: Crystal structure of glutamyl-tRNA synthetase from Helicobacter pylori
Source: Acta Crystallogr F Struct Biol Commun. 2024 Nov 27;80(Pt 12):335–40. doi: 10.1107/S2053230X24011099 (PMC11614106; doi:10.1107/S2053230X24011099)
Supplement: Supplementary file 1 [file f-80-00335-sup1.pdf]

STRUCTURAL BIOLOGY COMMUNICATIONS

Volume 80 (2024)

Supporting information for article:

Crystal structure of glutamyl-tRNA synthetase from *Helicobacter pylori*

Dylan E. Davis, Jesuferanmi P. Ayanlade, David T. Laseinde, Sandhya Subramanian, Hannah Udell, Donald J. Lorimer, David M. Dranow, Thomas E. Edwards, Peter J. Myler and Oluwatoyin A. Asojo

Figure S.1. ENDSCRIPT analysis reveals the nearest structural neighbors of *Hp*GluRS and shows extensive sequence conservation across multiple organisms. Identical and conserved residues are highlighted in red and yellow, respectively. The different secondary structure elements shown are alpha helices (α), 3_{10}-helices (η), beta strands (β), and beta turns (TT).

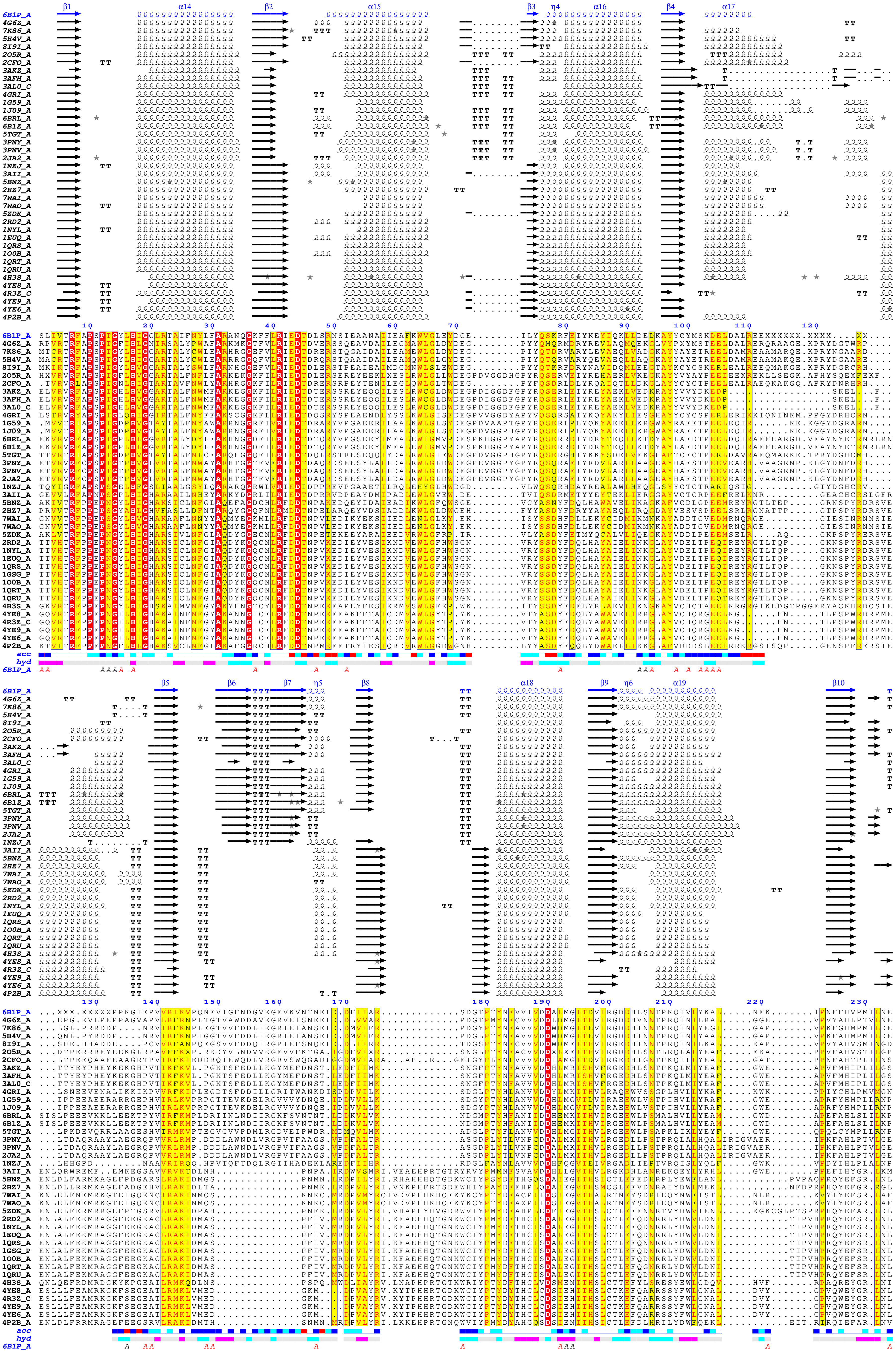

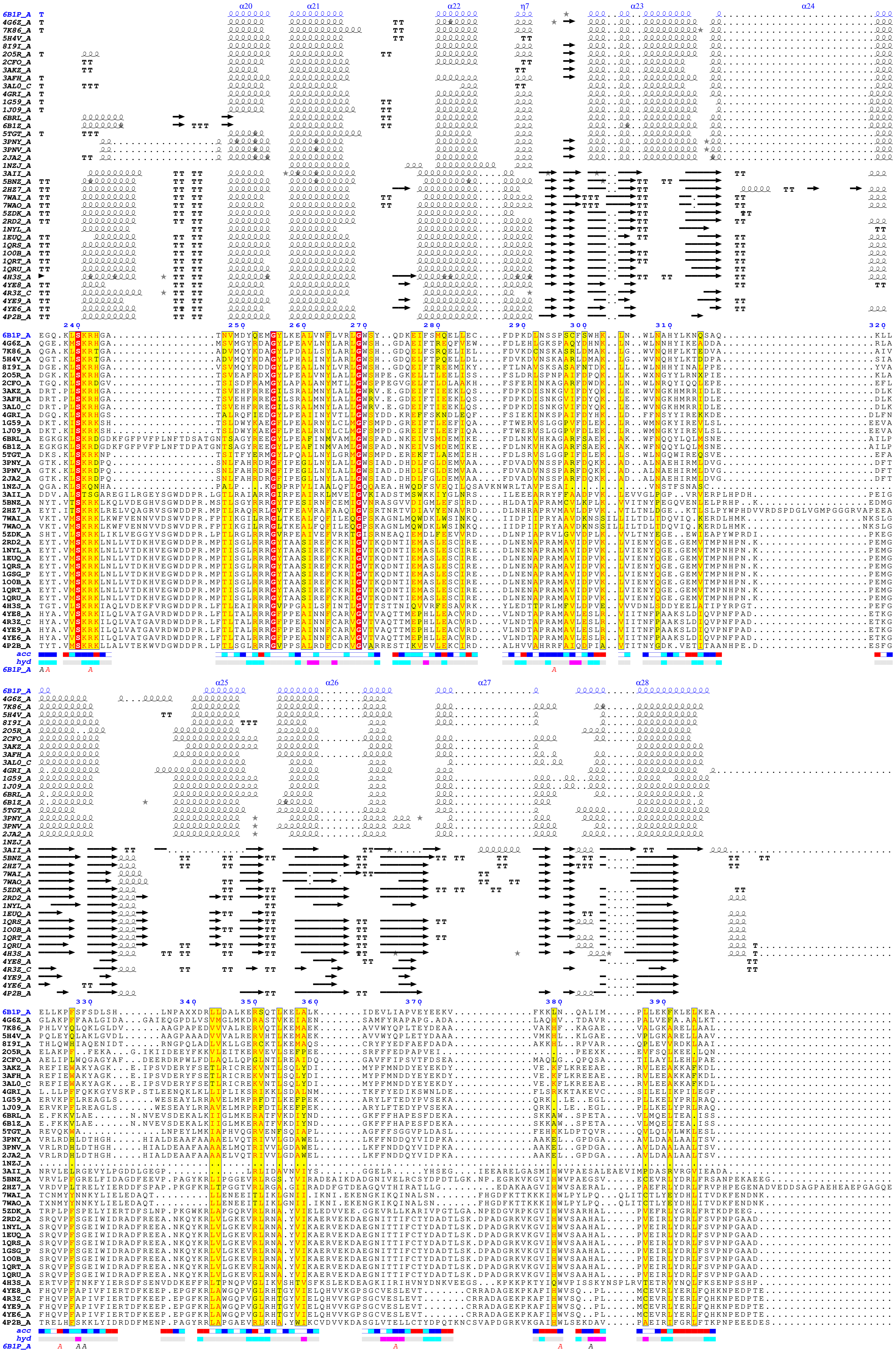

α20
α21
α22
η7
α23
α24
6B1P_A
4G6Z_A
7K86_A
5H4V_A
8I9I_A
2O5R_A
2CFO_A
3AKZ_A
3AFH_A
3AL0_C
4GRI_A
1G59_A
1J09_A
6BRL_A
6B1Z_A
5TGT_A
3PNY_A
3PNV_A
2JA2_A
1NZJ_A
3AII_A
5BNZ_A
2HZ7_A
7WAI_A
7WAO_A
5ZDK_A
2RD2_A
1NYL_A
1EUQ_A
1QRS_A
1GSG_P
1O0B_A
1QRT_A
1QRU_A
4H3S_A
4YE8_A
4R3Z_C
4YE9_A
4YE6_A
4P2B_A
acc
hyd
240
250
260
270
280
290
300
310
320
α25
α26
α27
α28
330
340
350
360
370
380
390

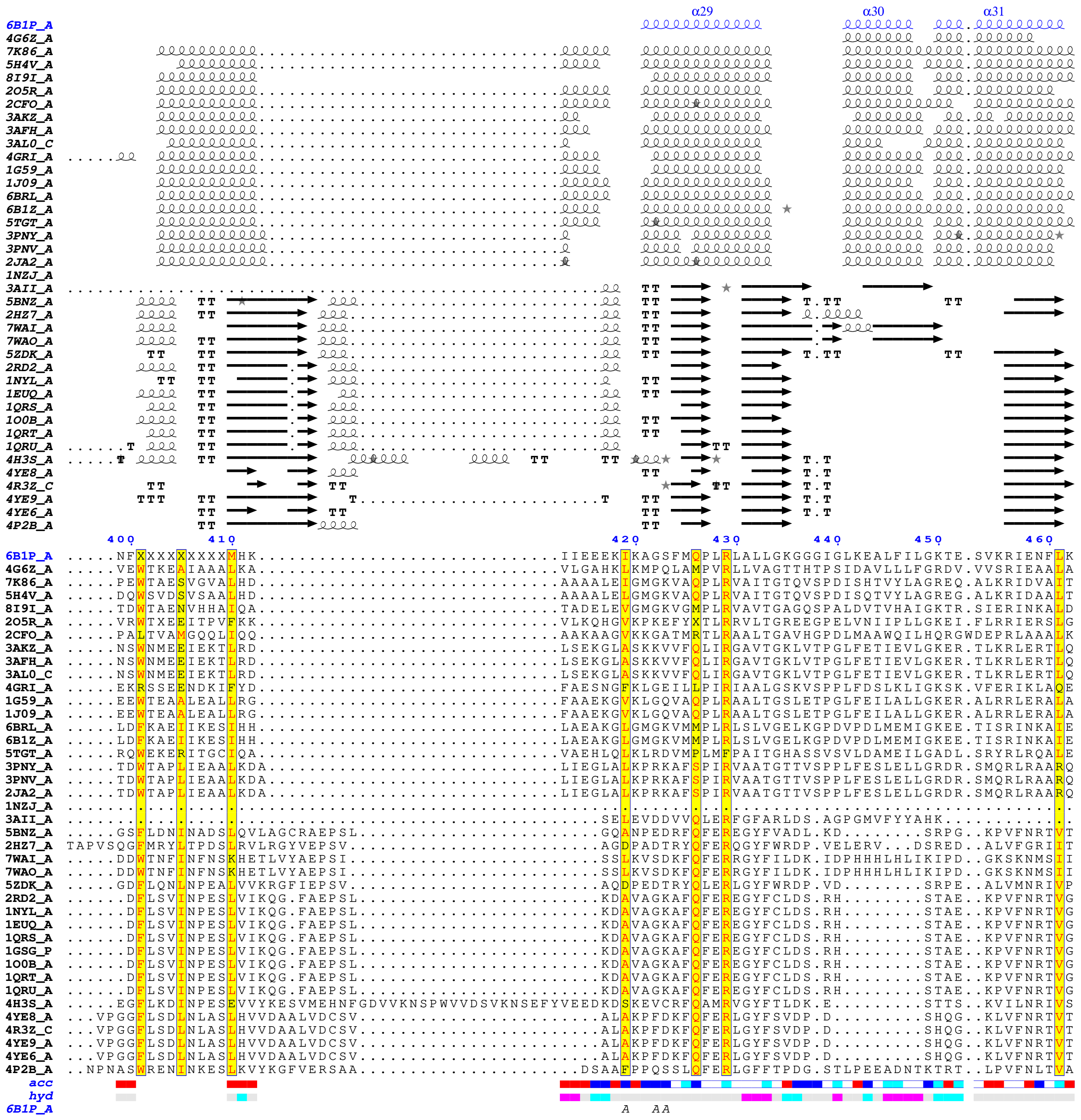

α29
α30
α31
400
410
420
430
440
450
460
acc
hyd

PDBe Fold v2.59. (src3) 14 Apr 2014 result file.

RESULT SUMMARY

##	Q-score	P-score	Z-score	RMSD	Nalgn	Nsse	Ngaps	Seq-%	Nmd	Nres-Q	Nsse-Q	Nres-T	Nsse-T	Query	Target
1	0.5482	21.34	14.37	1.947	396	25	17	0.3434	0	430	27	468	31	PDB 6b1p:A	PDB 1gln:A
2	0.5466	23.71	15.33	1.873	391	25	16	0.422	0	430	27	468	30	PDB 6b1p:A	PDB 2o5r:A
3	0.5196	26.29	15.92	1.941	386	24	19	0.3549	0	430	27	470	29	PDB 6b1p:A	PDB 2cv1:A
4	0.5193	25.1	15.58	1.934	385	24	20	0.3558	0	430	27	469	29	PDB 6b1p:A	PDB 2cv2:A
5	0.5165	22.34	14.93	2.002	388	24	23	0.3402	0	430	27	469	29	PDB 6b1p:A	PDB 1n78:A
6	0.5118	25.03	16.06	1.947	383	24	20	0.3629	0	430	27	469	29	PDB 6b1p:A	PDB 2dxi:A
7	0.5096	22.37	15.06	1.971	384	23	20	0.3542	0	430	27	470	28	PDB 6b1p:A	PDB 2cv0:A
8	0.5094	24.36	15.87	1.986	384	24	23	0.3646	0	430	27	468	29	PDB 6b1p:A	PDB 1n77:A
9	0.5037	23.7	15.07	1.955	380	24	19	0.3605	0	430	27	468	30	PDB 6b1p:A	PDB 1g59:A
10	0.4968	22.2	14.64	2.255	396	26	18	0.351	0	430	27	469	30	PDB 6b1p:A	PDB 1n78:B
11	0.4928	21.35	14.4	2.233	393	26	20	0.3461	0	430	27	469	31	PDB 6b1p:A	PDB 2cv2:B
12	0.49	19.48	14.22	2.122	383	26	16	0.4073	0	430	27	464	30	PDB 6b1p:A	PDB 3afh:A
13	0.4862	21.84	15.12	1.977	375	25	18	0.3573	0	430	27	469	30	PDB 6b1p:A	PDB 1j09:A
14	0.486	21.86	14.64	2.254	399	27	22	0.401	0	430	27	487	30	PDB 6b1p:A	PDB 2cfo:B
15	0.4857	21.56	15.04	2.015	377	25	19	0.3607	0	430	27	469	30	PDB 6b1p:A	PDB 2cuz:A
16	0.4834	15.49	13.09	2.236	396	25	20	0.3232	0	430	27	485	31	PDB 6b1p:A	PDB 3pnv:B
17	0.4826	22.17	15.23	1.920	370	25	16	0.3622	0	430	27	468	30	PDB 6b1p:A	PDB 1n75:A
18	0.4758	15.18	13.2	2.286	396	25	18	0.3207	0	430	27	485	32	PDB 6b1p:A	PDB 3pny:B
19	0.4748	21.75	15.09	2.155	381	24	22	0.3596	0	430	27	469	29	PDB 6b1p:A	PDB 2dxi:B
20	0.4727	14.74	12.57	2.362	398	26	21	0.3141	0	430	27	481	31	PDB 6b1p:A	PDB 3pnv:A
21	0.4719	20.55	14.76	2.131	378	24	21	0.3598	0	430	27	468	29	PDB 6b1p:A	PDB 1n77:B
22	0.4717	19.95	14.42	2.116	377	24	20	0.3607	0	430	27	468	31	PDB 6b1p:A	PDB 1g59:C
23	0.4716	20.1	14.59	2.249	385	24	18	0.3584	0	430	27	468	29	PDB 6b1p:A	PDB 2cv1:B
24	0.4699	19.56	14.66	1.971	332	22	13	0.4217	0	430	27	381	25	PDB 6b1p:A	PDB 4g6z:A
25	0.4687	13.39	12.29	2.311	386	23	18	0.3834	0	430	27	464	26	PDB 6b1p:A	PDB 3akz:A
26	0.4669	14.5	12.45	2.385	397	26	22	0.3073	0	430	27	481	31	PDB 6b1p:A	PDB 3pny:A
27	0.4669	20.67	14.79	2.165	378	24	21	0.3651	0	430	27	468	30	PDB 6b1p:A	PDB 2cv0:B
28	0.4654	20.49	14.26	2.447	402	26	21	0.403	0	430	27	485	30	PDB 6b1p:A	PDB 2cfo:A
29	0.4604	15.87	12.82	2.340	393	26	20	0.3155	0	430	27	485	30	PDB 6b1p:A	PDB 2ja2:A
30	0.4521	12.18	12.16	2.309	379	24	25	0.3879	0	430	27	464	30	PDB 6b1p:A	PDB 3akz:C
31	0.4237	10.86	11.58	2.259	364	22	17	0.3956	0	430	27	464	28	PDB 6b1p:A	PDB 3akz:B
32	0.3981	10.19	11.68	2.469	380	24	22	0.3474	0	430	27	503	33	PDB 6b1p:A	PDB 6brl:A
33	0.3912	12.76	12.31	2.594	378	25	19	0.3598	0	430	27	486	30	PDB 6b1p:A	PDB 4gri:B
34	0.3894	8.631	11.47	2.258	385	21	21	0.3766	0	430	27	565	29	PDB 6b1p:A	PDB 3al0:C
35	0.3844	14.72	13.17	2.723	380	24	18	0.3789	0	430	27	479	30	PDB 6b1p:A	PDB 4gri:A
36	0.3185	23.59	15.4	0.415	245	25	25	0.9918	0	430	27	430	28	PDB 6b1p:A	PDB 7k86:A

Supplementary Table S1.

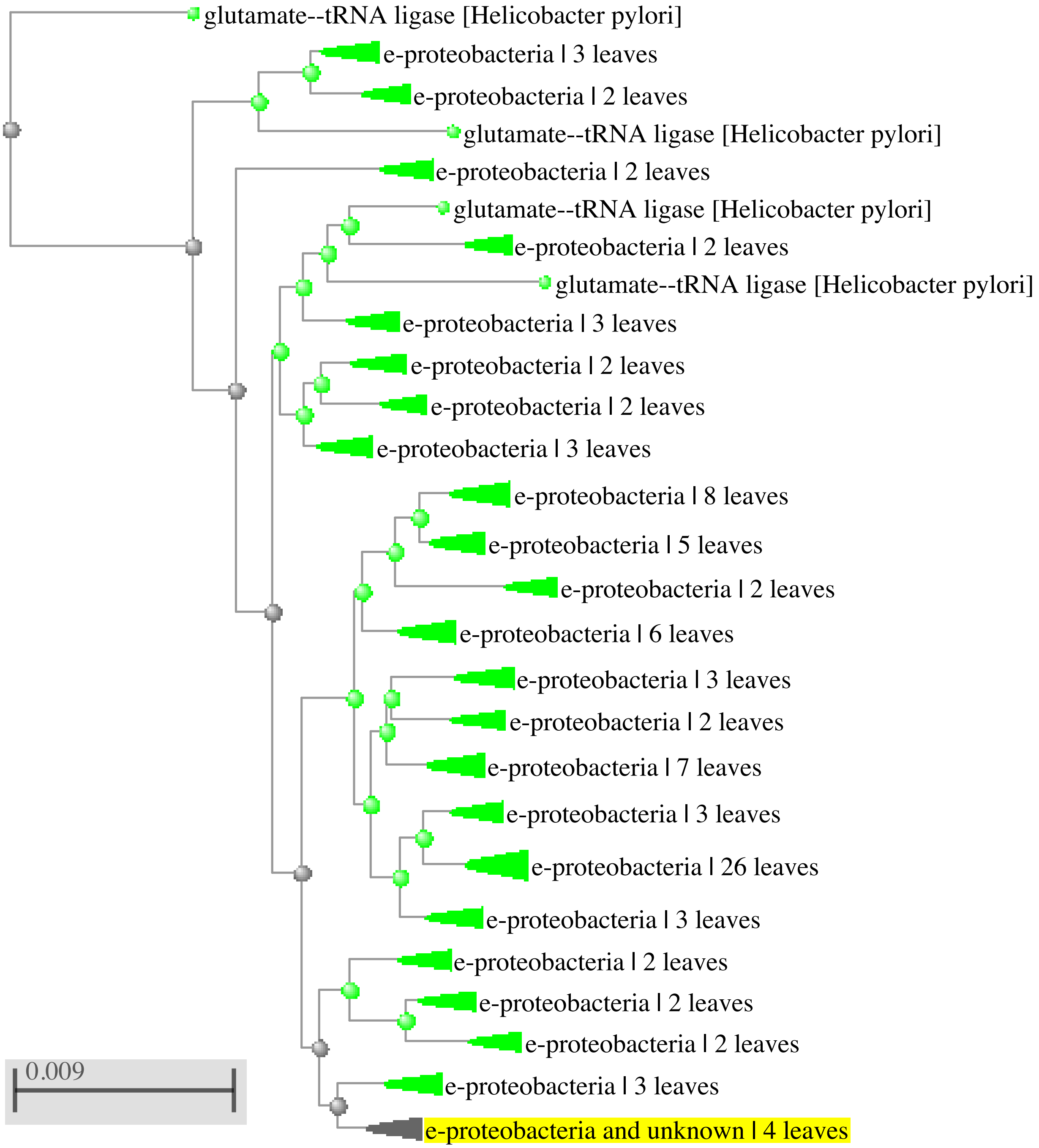

Figure S.2. Phylogenetic analysis of *Hp*GluRS and its nearest bacterial neighbors. The figure was generated using a protein blast against non-redundant sequences.

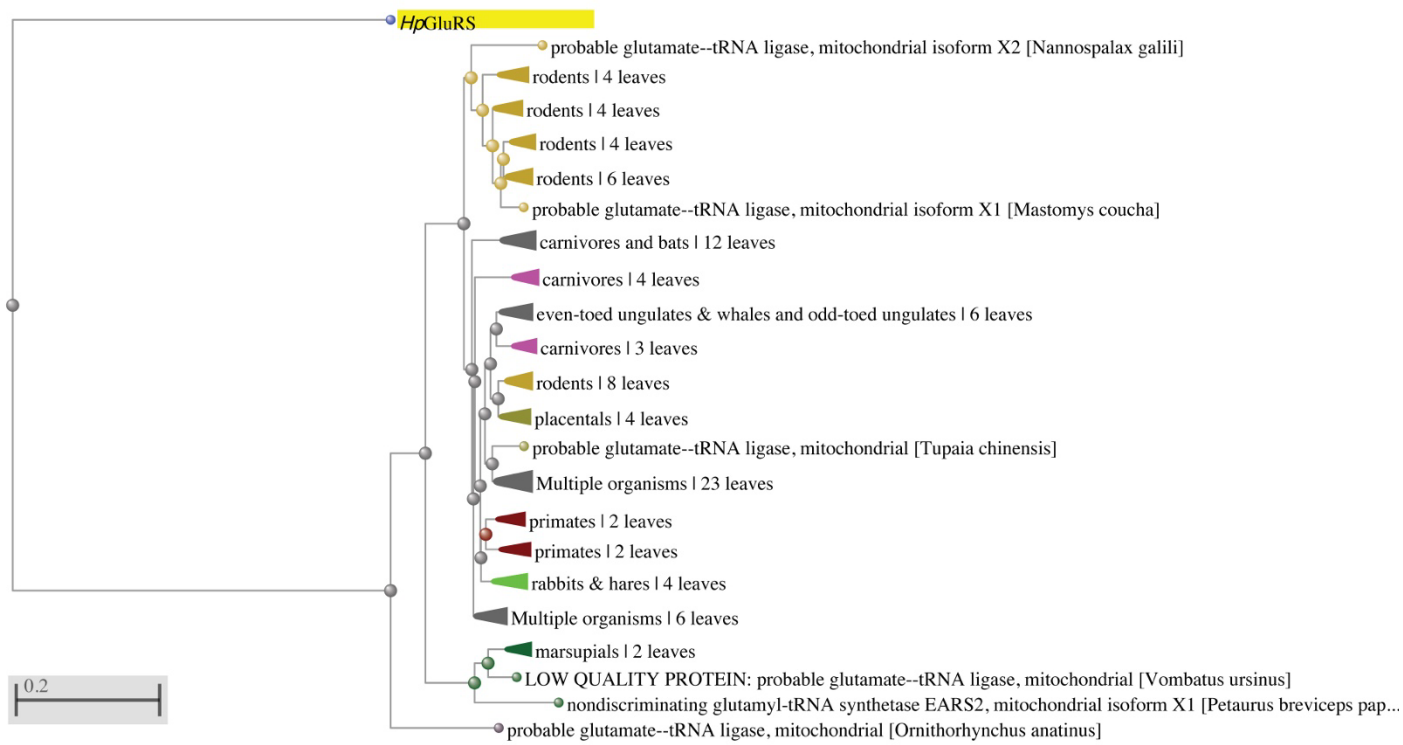

Figure S.3. Phylogenetic analysis of *Hp*GluRS and mammals. The figure was generated using a protein blast against all mammals.

Figure S.4. Alignment of *Hp*GluRS (PDB entry 6b1p) and its closest mammalian ortholog, human GluRS (PDB entry 4ye6). The identical residues are shown on a red background with conserved residues in red and conserved regions in blue boxes. Figure generated using ESPript 3.0 (Gouet *et al.*, 2003, Gouet *et al.*, 1999). The different secondary structure elements shown are alpha helices (α), 3_{10}-helices (η), beta strands (β), and beta turns (TT).

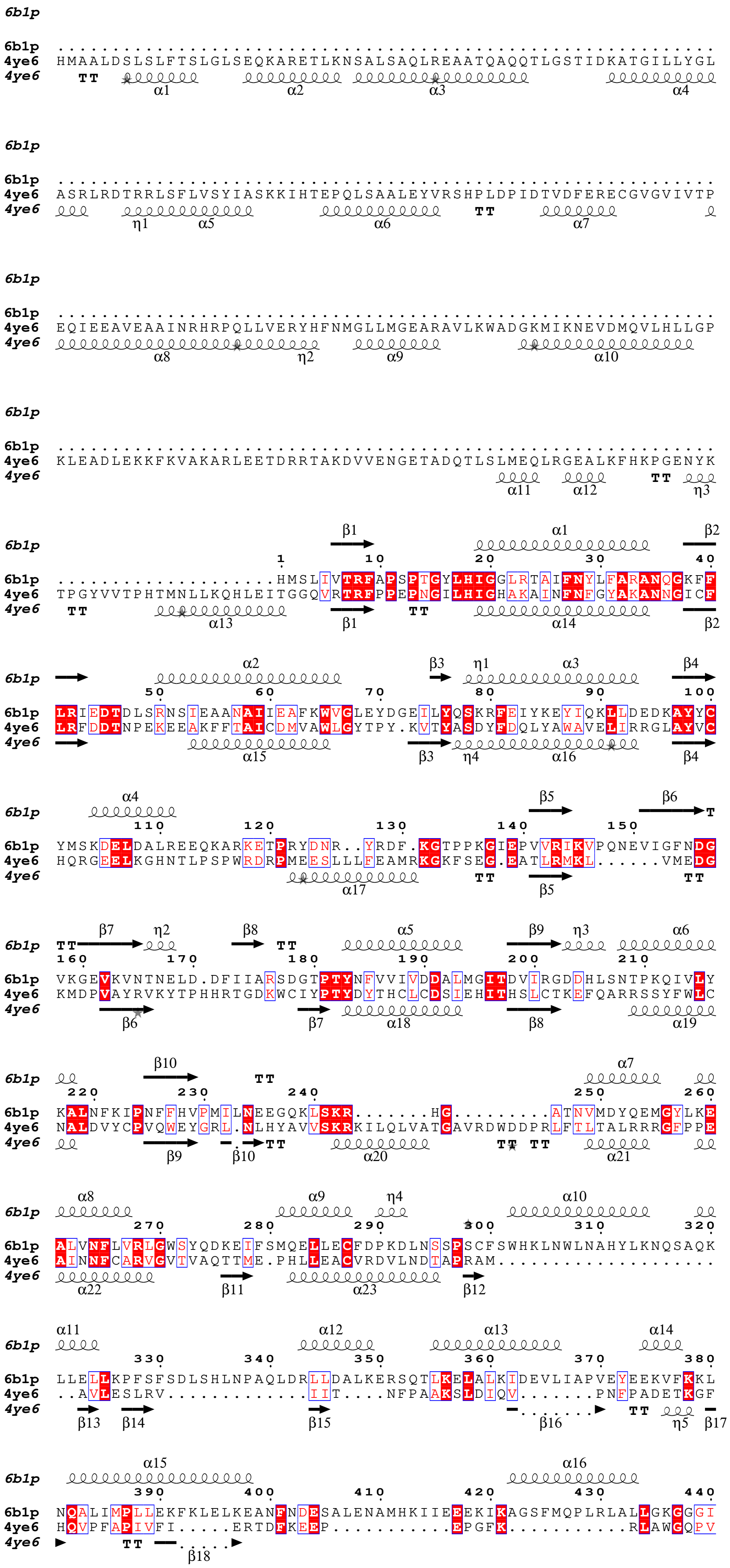
6b1p
4ye6 HMAALDSLSLFTSLGLSEQKARETLKNSALSAQLREAATQAQQTLGSTIDKATGILLYGL
4ye6 TT α1 α2 α3 α4
6b1p
4ye6 ASRLRDTRRLSFLVSYIASKKIHTEPQLSAALEYVRSHPLDPIDTVDFERECGVGVIVTP
4ye6 η1 α5 α6 TT α7
6b1p
4ye6 EQIEEAVEAAINRHRPQLLVERYHFNMGLLMGEARAVLKWADGKMIKNEVDMQVLHLLGP
4ye6 α8 η2 α9 α10
6b1p
4ye6 KLEADLEKKFKVAKARLEETDRRTAKDVVENGETADQTLSLMEQLRGEALKFHKPGENYK
4ye6 α11 α12 TT η3
6b1p β1 α1 β2
1 10 20 30 40
6b1p ...................HMSLIVTRFAPSPTGYLHIGGLRTAIFNYLFARANQGKFF
4ye6 TPGYVVTPHTMNLLKQHLEITGGQVRTRFPPEPNGILHIGHAKAINFNFGYAKANNGICF
4ye6 TT α13 β1 TT α14 β2
6b1p α2 β3 η1 α3 β4
50 60 70 80 90 100
6b1p LRIEDTDLSRNSIEAANAIIEAFKWVGLEYDGEILYQSKRFEIYKEYIQKLLDEDKAYYC
4ye6 LRFDDTNPEKEEAKFFTAICDMVAWLGYTPY.KVTYASDYFDQLYAWAVELIRRGLAYVC
4ye6 α15 β3 η4 α16 β4
6b1p α4 β5 β6 T
110 120 130 140 150
6b1p YMSKDELDALREEQKARKETPRYDNR..YRDF.KGTPPKGIEPVVRIKVPQNEVIGFNDG
4ye6 HQRGEELKGHNTLPSPWRDRPMEESLLLFEAMRKGKFSEG.EATLRMKL......VMEDG
4ye6 α17 TT β5 TT
6b1p TT β7 η2 β8 TT α5 β9 η3 α6
160 170 180 190 200 210
6b1p VKGEVKVNTNELD.DFIIARSDGTPTYNFVVIVDDALMGITDVIRGDDHLSNTPKQIVLY
4ye6 KMDPVAYRVKYTPHHRTGDKWCIYPTYDYTHCLCDSIEHITHSLCTKEFQARRSSYFWLC
4ye6 β6 β7 α18 β8 α19
6b1p β10 TT α7
220 230 240 250 260
6b1p KALNFKIPNFFHVPMILNEEGQKLSKR.......HG.........ATNVMDYQEMGYLKE
4ye6 NALDVYCPVQWEYGRL.NLHYAVVSKRKILQLVATGAVRDWDDPRLFTLTALRRRGFPPE
4ye6 β9 β10 TT α20 TT TT α21
6b1p α8 α9 η4 α10
270 280 290 300 310 320
6b1p ALVNFLVRLGWSYQDKEIFSMQELLECFDPKDLNSSPSCFSWHKLNWLNAHYLKNQSAQK
4ye6 AINNFCARVGVTVAQTTME.PHLLEACVRDVLNDTAPRAM....................
4ye6 α22 β11 α23 β12
6b1p α11 α12 α13 α14
330 340 350 360 370 380
6b1p LLELLKPFSFSDLSHLNPAQLDRLLDALKERSQTLKELALKIDEVLIAPVEYEEKVFKKL
4ye6 ..AVLESLRV............IIT....NFPAAKSLDIQV.......PNFPADETKGF
4ye6 β13 β14 β15 β16 TT η5 β17
6b1p α15 α16
390 400 410 420 430 440
6b1p NQALIMPLLEKFKLELKEANFNDESALENAMHKIIEEEKIKAGSFMQPLRLALIGKGGGI
4ye6 HQVPFAPIVFI.....ERTDFKEEP...........EPGFK...........RLAWGQPV
4ye6 TT β18

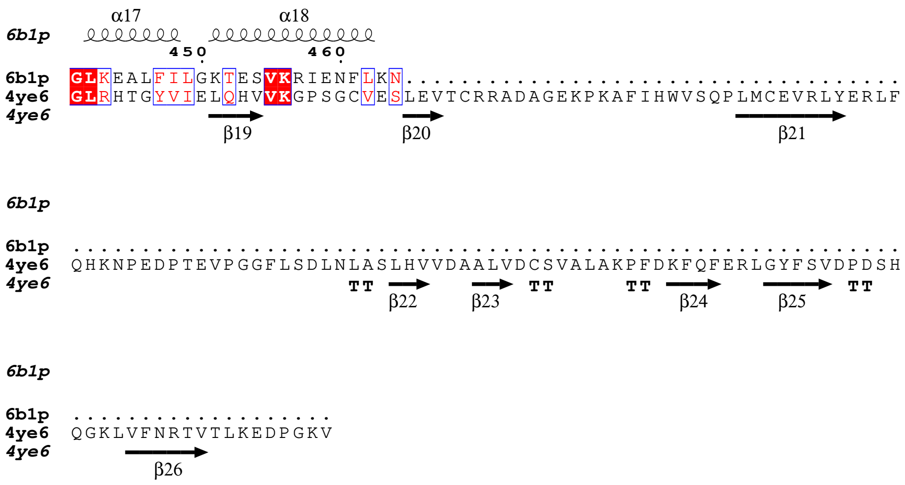
α17
α18
6b1p
450
460
6b1p GLKEALFILGKTESVKRIENFLKN
4ye6 GLRHTGYVIELQHVVKGPSGCVESLEVTCRRADAGEKPKAFIHWVSQPLMCEVRLYERLF
4ye6
β19
β20
β21
6b1p
4ye6 QHKNPEDPTEVPGGFLSDLNLASLHVVDAALVDCSVALAKPFDKFQFERLGYFSVDPDSH
4ye6
TT
β22
β23
TT
TT
β24
β25
TT
6b1p
4ye6 QGKLVFNRTVTLKEDPGKV
4ye6
β26
